# Reduced HIV/AIDS diagnosis rates and increased AIDS mortality due to late diagnosis in Brazil during the COVID-19 pandemic

**DOI:** 10.1038/s41598-023-50359-y

**Published:** 2023-12-27

**Authors:** Lucas Almeida Andrade, Thiago de França Amorim, Wandklebson Silva da Paz, Mariana do Rosário Souza, Emerson Lucas S. Camargo, Débora dos Santos Tavares, Shirley Verônica M. A. Lima, Enaldo Vieira de Melo, Marco Aurélio de O. Góes, Rodrigo Feliciano do Carmo, Carlos Dornels F. de Souza, Allan Dantas dos Santos, Álvaro Francisco L. de Sousa, Isabel Amélia C. Mendes, Abelardo Silva-Júnior, Wagnner José N. Porto, Márcio Bezerra-Santos

**Affiliations:** 1 Health Science Graduate Program, Universidade Federal de Sergipe, Aracaju, SE Brazil; 2 Graduate Program in Teaching and Teacher Training, Universidade Federal de Alagoas, Arapiraca, AL Brazil; 3 Tropical Medicine Graduate Program, Universidade Federal de Pernambuco, Recife, PE Brazil; 4 Ribeirão Preto College of Nursing, Universidade de São Paulo, Ribeirão Preto, SP Brazil; 5 Department of Health Science, Universidade Federal de Sergipe, Lagarto, SE Brazil; 6 Department of Nursing, Universidade Federal de Sergipe, Lagarto, SE Brazil; 7 Department of Medicine, Universidade Federal de Sergipe, Aracaju, Brazil; 8 College of Pharmaceutical Sciences, Federal University of Vale do São Francisco, Petrolina, PE Brazil; 9 College of Medicine, Federal University of Vale do São Francisco, Petrolina, PE Brazil; 10 Institute of Teaching and Research, Hospital Sírio-Libânes, São Paulo, SP Brazil; 11 National School of Public Health, Public Health Research Centre, Comprehensive Health Research Center, REAL, NOVA University of Lisbon, Lisbon, Portugal; 12 Health Science Graduate Program, Universidade Federal de Alagoas, Maceió, AL Brazil; 13 Institute of Biological and Health Sciences, Universidade Federal de Alagoas, Maceió, AL Brazil; 14 Animal Sciences Graduate Program, Universidade Federal de Alagoas, Maceió, AL Brazil; 15 Medical and Nursing Science Complex, Universidade Federal de Alagoas, Arapiraca, AL Brazil

**Keywords:** Infectious diseases, HIV infections

## Abstract

The COVID-19 pandemic has severely affected global health, leading to the suspension of numerous routine healthcare services and posing challenges in efforts to control other diseases, such as HIV/AIDS. This study aimed to assess the impact of the COVID-19 pandemic on HIV/AIDS diagnoses and mortality rates in Brazil during 2020 and 2021. The percentage change was calculated to determine whether there was an increase or decrease in HIV/AIDS diagnoses and mortality, considering the average numbers from the last 5 years. Additionally, a Joinpoint regression model and an interrupted time series analysis were applied to assess time trends before and after the onset of the pandemic. Lastly, choropleth maps were prepared. We observed a reduction of 22.4% (2020) and 9.8% (2021) in the diagnosis of HIV/AIDS in Brazil. Conversely, there was a significant increase in the percentage change of late diagnosis of AIDS deaths in 2020 (6.9%) and 2021 (13.9%), with some states showing an increase of over 87%. Decreasing time trends in the diagnosis of HIV/AIDS were identified before the pandemic in Brazil, especially in the Southeast and South regions, and then time trends stabilized after including the pandemic years. Along with the dissemination of COVID-19, there was a reduction in the diagnosis of HIV/AIDS and an increase in late diagnosis AIDS deaths, signaling a serious impact of the pandemic on HIV/AIDS control strategies in Brazil. Therefore, we highlight the need for continuous efforts to control both diseases, that is, maintaining regular health services even in crisis situations.

## Introduction

In recent decades, humanity has faced two of the most severe viral epidemics in history. The first, caused by the human immunodeficiency virus (HIV), emerged in the early 1980s, resulting in approximately 32 million deaths from the acquired immunodeficiency syndrome (AIDS)^1^. The second, caused by the severe acute respiratory syndrome coronavirus 2 (SARS-CoV-2), emerged in December 2019 in Wuhan, China, and was declared a pandemic by the World Health Organization (WHO) in March 2020. As of now, Coronavirus disease 2019 (COVID-19) has been responsible for approximately 6.8 million deaths worldwide, severely impacting public health and the global economy^2,3^.

HIV/AIDS has been one of the most devastating pandemics in history. Even after 40 years since HIV was first identified, AIDS remains a major global public health issue, with about 75 million people diagnosed and almost half of them dying from the disease^4,5^. In 2019, the Joint United Nations Program on HIV/AIDS (UNAIDS) estimated that 38 million people were living with HIV, with 1.7 million new infections and 690,000 related deaths that year. Over 400,000 AIDS-related deaths were recorded in Africa alone, accounting for more than half of the global mortality rate. Compared to 2010, HIV incidence in 2019 decreased by 23% and mortality by 37%, but the number of people living with HIV was 24% higher in 2019 than in 2010^6,7^.

Despite advances in prevention, HIV testing, and the continuous use of antiretroviral therapy (ART), the HIV pandemic continues to expand in Eastern Europe, Central Asia, the Middle East, Africa, and Latin America^5–11^. Latin America, in particular, has the second-highest HIV prevalence rate globally, with an estimated 2.2 million [1.5 million–2.8 million] people living with HIV/AIDS. The region has not seen a reduction in infection rates over the last decade. Brazil plays a significant role in this scenario due to its large population and high concentration of cases, accounting for 35% of the total population living with HIV and 47% of new infections in the region^12^.

In Brazil, the Ministry of Health reported that, in 2018, there were approximately 43,941 new cases of HIV infection and 37,161 new AIDS patients. However, there was a decrease in the incidence of opportunistic infections, hospitalization rates, and AIDS mortality rate (a 22.8% reduction between 2014 and 2018) in the country^12^. Conversely, there was an increase in infection rates among young people aged 15 to 24 years. Despite control strategies like prevention campaigns, early diagnosis, and timely treatment, HIV/AIDS remains a global challenge, and the goal of controlling the disease seems far from being achieved^12,13^.

Meanwhile, the pandemic caused by SARS-CoV-2 is considered, to date, the greatest global public health challenge of this millennium^14^. COVID-19, primarily a respiratory viral infection, has a fatality rate ranging from 0.1 to 25%^15^. To reduce the spread of SARS-CoV-2 and avoid overwhelming healthcare systems, governments and health authorities have implemented emergency measures. These include social distancing and/or isolation, reallocating healthcare professionals to pandemic response, suspending non-emergency outpatient services, and temporarily halting programs for the control, prevention, and treatment of other diseases^14–19^, including HIV/AIDS^20^.

Previous studies have shown that during the pandemic, there was a significant reduction in the diagnoses of tropical diseases such as leprosy^21^, tuberculosis^22^, and schistosomiasis^23^. Additionally, the pandemic has negatively impacted the diagnosis of hepatitis C virus in Brazil^24^. Consequently, this study aims to assess the impact of the COVID-19 pandemic on the diagnosis of HIV/AIDS and its related mortality in all Brazilian states during the first two years of the pandemic (2020 and 2021).

## Material and methods

### Type and study design

An ecological, population-based study employing spatiotemporal techniques was conducted, utilizing data from the Brazilian Ministry of Health’s Notifiable Diseases Information System (SINAN) from 2015 to 2021. To assess the expected number of HIV/AIDS cases and deaths during 2020 and 2021, we calculated the average number of cases over the previous five years (2015 to 2019 for 2020; and 2016 to 2020 for 2021). However, to address the potential decrease in the expected average for 2021 due to the observed cases in 2020, we used the expected value for 2020. This approach provided a more reliable estimate of the expected average for the period from 2016 to 2020. Through this methodology, we could effectively evaluate the impact of the COVID-19 pandemic on the reporting of HIV/AIDS cases and related deaths across all Brazilian states.

### Study area

**Figure 1:**
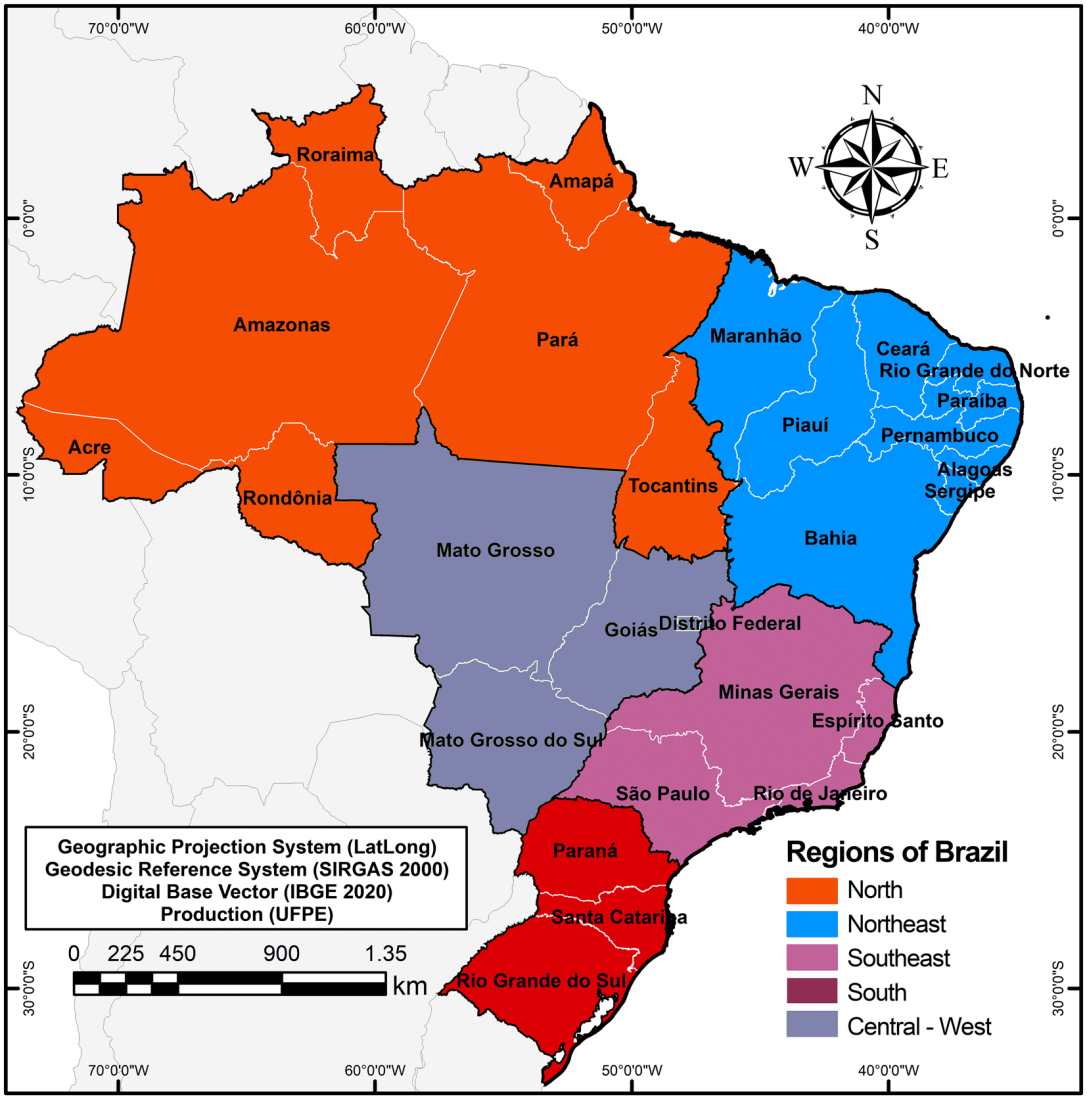
Study area: map of Brazil divided into five regions, and its 26 states and one Federal District. QGis *3.20.4* (https://qgis.org/pt_BR/site/).

Brazil, a continental country located in South America, is the fifth-largest country in the world by area. It has a population of approximately 212 million, making it the sixth most populous country globally. Politically and administratively, Brazil is divided into 26 states and one Federal District, encompassing 5570 municipalities. For political and operational purposes, these states are grouped into five regions: North, Northeast, Southeast, South, and Central-West, each with distinct geographic and cultural features (Fig. 1)^25^. Despite being the 12th largest economy in the world, with a gross domestic product of about US$ 1.434 trillion in 2021, Brazil faces significant social inequalities across its regions and states. Additionally, it is endemic for several Neglected Tropical Diseases (NTDs) such as Leishmaniasis^26^, Schistosomiasis^27,28^, Dengue^29^, Chagas disease^30^, and Leprosy^31^.

### Variables and data sources

The variables used in this study included: (i) the total number of HIV/AIDS diagnoses; (ii) late diagnosis AIDS deaths (referring to patients who had not been tested for and/or were unaware of their HIV serological status and were only diagnosed with AIDS at the time of death); and (iii) the total number of deaths from AIDS. Data for these variables were collected from three sources: the Notifiable Diseases Information System (SINAN), the Mortality Information System (SIM), and the Control System for Laboratory Tests (SISCEL), all managed by the Brazilian Ministry of Health. It is important to note that the notification of HIV/AIDS cases is mandatory across Brazil. The data from SINAN, SIM, and SISCEL are publicly available and can be accessed on the website of the Information Technology Department of the Unified Health National System (DATASUS). Additionally, the Brazilian digital cartographic mesh, divided by states and regions, was obtained in shapefile format (Geodetic Reference System, SIRGAS/2000) from the website of the Brazilian Institute of Geography and Statistics (IBGE)^25^.

### Data analysis and percentage change calculation

To assess the impact of the COVID-19 pandemic on the number of HIV/AIDS diagnoses, late AIDS diagnosis, and its related mortality across all Brazilian states, we calculated the percentage change based on data from SINAN, SIM, and SISCEL. This method of calculating percentage change has been utilized in previous studies on diseases such as leprosy^21^, tuberculosis^22^, and schistosomiasis^23^, to analyze morbidity rates and other indicators. By comparing the expected and observed values, it’s possible to evaluate the increase or decrease in disease occurrence and/or mortality over time and across different locations^32^.

The expected values for the study variables in 2020 and 2021 were determined based on the average number of registered cases/deaths over the preceding five years (2015 to 2019 for 2020, and 2016 to 2020 for 2021). These expected values were then compared with the actual observed values during each year of the pandemic. This comparison facilitated the calculation of the percentage change for 2020 and 2021. Consequently, positive percentage change values indicate an increase, while negative values suggest a decrease in the number of diagnoses or deaths compared to the expected values. The percentage change was analyzed at the regional, state, and national levels. The results were presented in bar graphs, illustrating the percentage change in total HIV/AIDS diagnoses and late diagnoses related to AIDS mortality in Brazil, during 2020 and 2021. However, it is important to note that data related to the total number of AIDS deaths were only available up to 2020.

### Temporal trend analysis

A segmented log-linear regression, utilizing the joinpoint regression model, was employed to assess the temporal trend of AIDS diagnosis and mortality rates. The selection of the best model, based on inflection points, was determined using the Monte Carlo permutation test with 999 permutations. In this analysis, the number of AIDS diagnoses and deaths were treated as dependent variables, while the years were considered as independent variables^33^.

Additionally, to describe and quantify these temporal trends, annual percentage changes (APCs) and their respective 95% confidence intervals (CIs) were calculated. When multiple significant inflection points were identified during the study period, average annual percentage changes (AAPCs) were also computed. Temporal trends were deemed statistically significant if the APCs had a p-value less than 0.05 and their 95% CIs did not encompass zero. A positive and significant APC indicated an increasing trend, while a negative and significant APC pointed to a decreasing trend. Trends without statistical significance were described as stable, regardless of the APC values^33^.

### Interrupted time series analyses

To evaluate whether the total number of AIDS deaths in 2020, following the onset of COVID-19 in Brazil, deviated from the trend observed between 2015 and 2019, an interrupted time series analysis was conducted. Unfortunately, only the data on total AIDS deaths were available on a monthly basis from SIM. Data on the total number of HIV/AIDS diagnoses and late diagnoses related to AIDS mortality were provided annually, precluding the possibility of conducting an interrupted time series analysis for these variables. In the analysis, the intervention model was defined as the onset of the COVID-19 pandemic in Brazil in March 2020. Initially, graphs of residue, sample, and partial autocorrelation functions (ACF and partial ACF) were utilized to check for autocorrelation in the residue and to assess stationarity and normality properties, aiding in selecting the most suitable and statistically parsimonious models^34^. Subsequently, ARIMA models of serial dependence were identified. The selected pre-intervention model was an ARIMA (2, 1, 0). The Ljung-Box (Q) test was then applied to evaluate whether the residuals were white noise, i.e. approximately normally distributed around zero^35^. This test verifies if the models adequately describe the linear dependence between successive data points.

### Spatial analyses and elaboration of choropleth maps

To assess the spatial distribution of the data, choropleth maps were created, depicting the percentage change values by Brazilian states for the years 2020 and 2021. These maps included data on: (i) the total number of HIV/AIDS diagnoses; (ii) late diagnosis AIDS deaths; (iii) total deaths from AIDS. The maps were divided into nine categories of equal intervals based on the percentage change values (positive or negative). Additionally, states were classified according to the following percentage change intervals: − 100 to − 75%; − 74.99 to − 50%; − 50 to − 25%; − 24.99 to − 0.1%; 0%; 0.00 to 25%; 25.01 to 50%; 50.01 to 75%; and ≥ 75%.

### Software utilized

For the analysis of percentage change and graph construction, Microsoft Office Excel 2019 was employed. The Joinpoint Regression Program v. 4.5.0 (Kim et al. 2000; NCI, 2013) was utilized to calculate temporal trends. Interrupted time series analyses were conducted using IBM SPSS Statistics 22 software. Finally, QGIS 3.20.4 (QGIS Development Team—Open Source Geospatial Foundation Project) was used for generating the choropleth maps.

### Ethical considerations

The data utilized in this study is publicly available and does not contain any personally identifiable information. Therefore, obtaining individual consent forms was not necessary, nor was approval by an Ethics and Research Committee mandatory. However, the principles of the Helsinki Convention were adhered to throughout the study.

## Results

**Figure 2:**
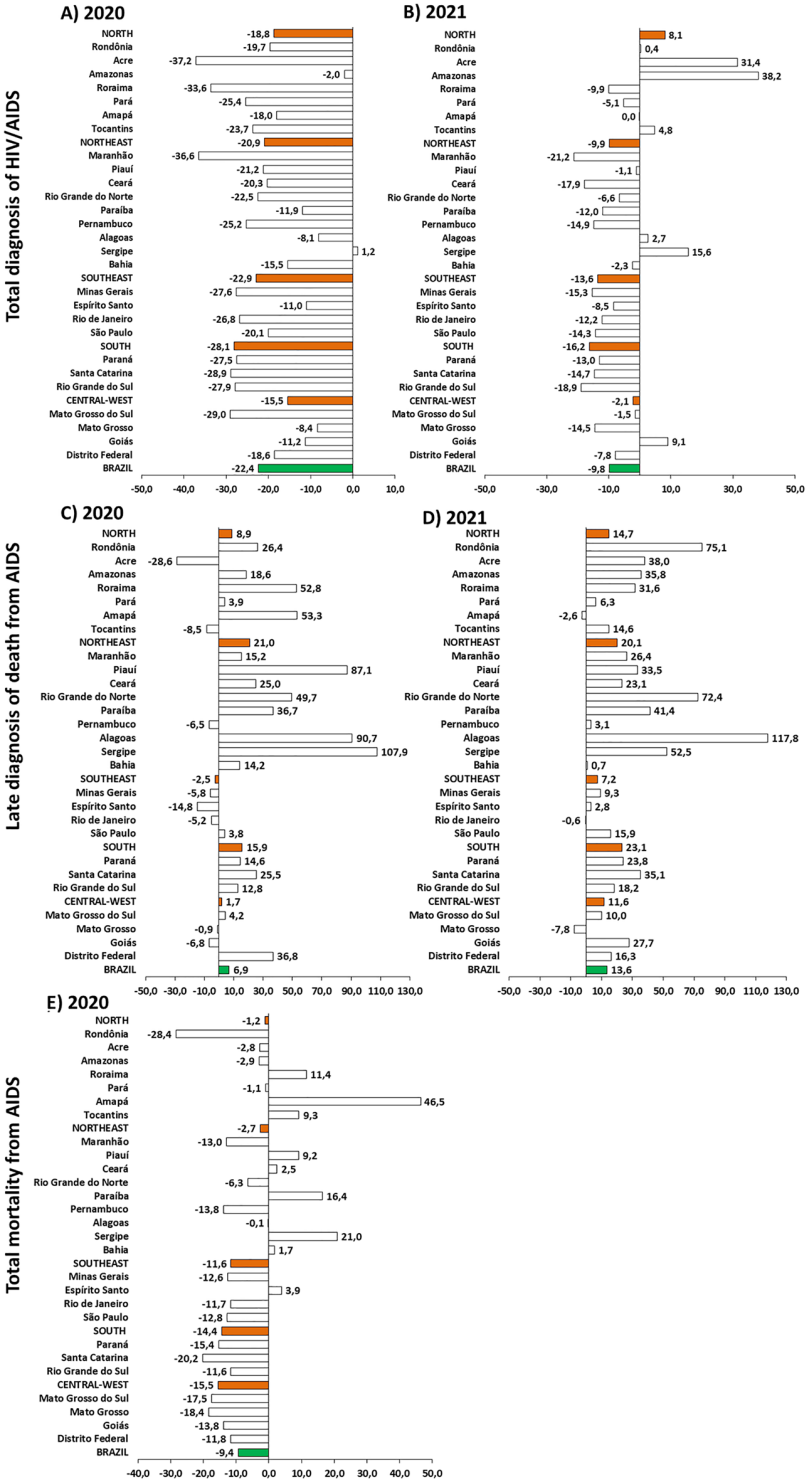
Percentage change in key indicators of AIDS diagnosis and related mortality in Brazilian states, regions, and country during 2020 and 2021. (**A**) Number of HIV/AIDS cases in Brazil in 2020; (**B**) Number of HIV/AIDS cases in Brazil in 2021; (**C**) Late diagnosis AIDS deaths in Brazil in 2020; (**D**) Late diagnosis AIDS deaths in Brazil in 2021; (**E**) Total number of AIDS deaths in Brazil in 2020.

According to the analysis, there was a reduction of 8858.8 in the total number of HIV/AIDS diagnoses in Brazil during 2020 (n = 30,638; percentage change = − 22.4%) compared to the average number of cases reported in the previous five years (2015 to 2019, n = 39,496.8; Fig. 2A). In 2021, this reduction was 3846.36 (percentage change = − 9.8%; Fig. 2B). Similarly, all Brazilian states (except Sergipe) and regions showed a reduction in the number of HIV/AIDS cases during 2020. The South region (− 28.1%) and the states of Acre (− 37.2%), Maranhão (-36.6%), and Roraima (− 33.6%) had the highest percentages of reduction in the number of HIV/AIDS cases diagnosed in 2020. Conversely, the North region (8.1%) and states such as Amazonas (38.2%), Acre (31.4%), and Sergipe (15.6%) showed increases in HIV/AIDS diagnoses in 2021. However, most Brazilian states and regions continued to experience a decrease in the number of HIV/AIDS diagnoses in 2021.

More notably, there was a significant increase in the percentage of late diagnosis AIDS deaths in both years (2020 and 2021) (Fig. 2C and D). In Brazil, these increases were 6.9% and 13.9%, respectively. The states of Sergipe (107.9%), Alagoas (90.7%), and Piauí (87.1%) showed the highest percentage increases in 2020. Similarly, Alagoas exhibited a percentage increase of 117.8% during 2021. On the other hand, there was a decrease in the overall number of AIDS deaths in Brazil during 2020 (− 9.4%; Fig. 2E). This reduction in the percentage change was observed in all regions and most states of the country. The largest decreases were observed in the states of Rondônia (− 28.4%), Santa Catarina (− 20.2%), and Mato Grosso (− 18.4%). In contrast, the states of Amapá (46.5%), Sergipe (21%), and Paraíba (16.4%) showed the highest percentage increases in total AIDS mortality in Brazil during 2020.

Additionally, the temporal trend analyses of data and the annual percentage change (APC) of the number of AIDS cases, late diagnosis AIDS deaths, and the total number of deaths from AIDS in Brazil were calculated (Table 1). Notably, the number of AIDS cases during the years before the pandemic (2015–2019) presented a decreasing trend in Brazil (APC = − 1.9%; p-value = 0.012) and also in the Southeast (APC = − 4.9%; p-value < 0.001) and South (APC = − 4.1%; p-value = 0.016) regions. However, time trends including the pandemic years (2015–2021) remained stable in Brazil and its regions.

**Table 1:** Temporal trend analyses of the number of HIV/AIDS cases, late diagnosis AIDS deaths and total AIDS deaths in Brazil and its regions, during 2015–2019, and 2015–2021.

Variables	2015–2019				2015–2020^#^ or 2021			
	APC	CI 95%	Trend	p-value	APC	CI 95%	Trend	p-value
Total diagnosis of HIV/AIDS								
Brazil	− 1.9*	− 3.1 to − 0.8	Decreasing	0.012	− 3.4	− 11.4 to 5.4	Stable	0.435
North	2.5	− 1.6 to 6.7	Stable	0.153	0.9	− 13.9 to 18.2	Stable	0.914
Northeast	0.7	− 0.6 to 2.1	Stable	0.192	− 2.2	− 10.8 to 7.1	Stable	0.629
Southeast	− 4.9*	− 4.6 to − 3.7	Decreasing	< 0.001	− 4.8	− 11.2 to 2.1	Stable	0.168
South	− 4.1*	− 8 to − 1.7	Decreasing	0.016	− 5.8	− 14.2 to 3.4	Stable	0.208
Central-West	2.8	− 0.7 to 6.3	Stable	0.083	− 0.4	− 2.9 to 2.1	Stable	0.743
Late diagnosis AIDS deaths								
Brazil	− 2.0*	− 3.3 to − 0.7	Decreasing	0.017	1.7*	0.1 to 3.4	Increasing	0.039
North	2.8	− 1.6 to 7.4	Stable	0.136	2.1	− 6.4 to 11.3	Stable	0.638
Northeast	0.8	− 0.6 to 2.3	Stable	0.178	2.8	− 4.5 to 10.8	Stable	0.461
Southeast	− 4.2*	− 4.8 to − 3.5	Decreasing	< 0.001	− 0.4	− 2.6 to 1.8	Stable	0.71
South	− 5.5*	− 8.8 to − 2	Decreasing	0.016	5.0	0.4 to 10.7	Increasing	0.05
Central-West	3.1	− 1.1 to 7.5	Stable	0.101	1.3	− 6.7 to 10	Stable	0.757
Total of deaths from AIDS								
Brazil	− 4.4*	− 5.9 to − 2.8	Decreasing	0.003	− 3.9*	− 5.1 to − 2.7	Decreasing	0.001
North	− 0.1	− 3.7 to 3.7	Stable	0.94	− 0.2	− 2.3 to 1.9	Stable	0.76
Northeast	− 2.4*	− 4.0 to − 0.8	Decreasing	0.017	− 1.8*	− 3.1 to − 0.4	Decreasing	0.023
Southeast	− 6.3*	− 8.6 to − 3.9	Decreasing	0.004	− 5.4*	− 7.4 to − 3.3	Decreasing	0.002
South	− 5.3*	− 6.8 to − 3.9	Decreasing	0.002	− 5.2*	− 6.1 to − 4.3	Decreasing	< 0.001
Central-West	− 2.6*	− 3.8 to − 1.4	Decreasing	0.006	− 3.7*	− 5.6 to − 1.7	Decreasing	0.007

*p-value < 0.05; ^#^Data on total of deaths from AIDS was only available until 2020.

Regarding late diagnosis AIDS deaths, a decreasing trend was observed before the pandemic in Brazil (APC = − 2.0%; p-value = 0.017), in the Southeast (APC = − 4.2%; p-value < 0.001), and the South (APC = − 5.5%; p-value = 0.016) regions. The other regions showed stable trends during this period. Nonetheless, when data from 2020 and 2021 were included, the time trends became increasing in Brazil (APC = 1.7%; p-value = 0.039) and in the Southern region (APC = 5.0%; p-value = 0.05). The time trends for total AIDS deaths, however, did not change significantly with the onset of the pandemic.

**Figure 3:**
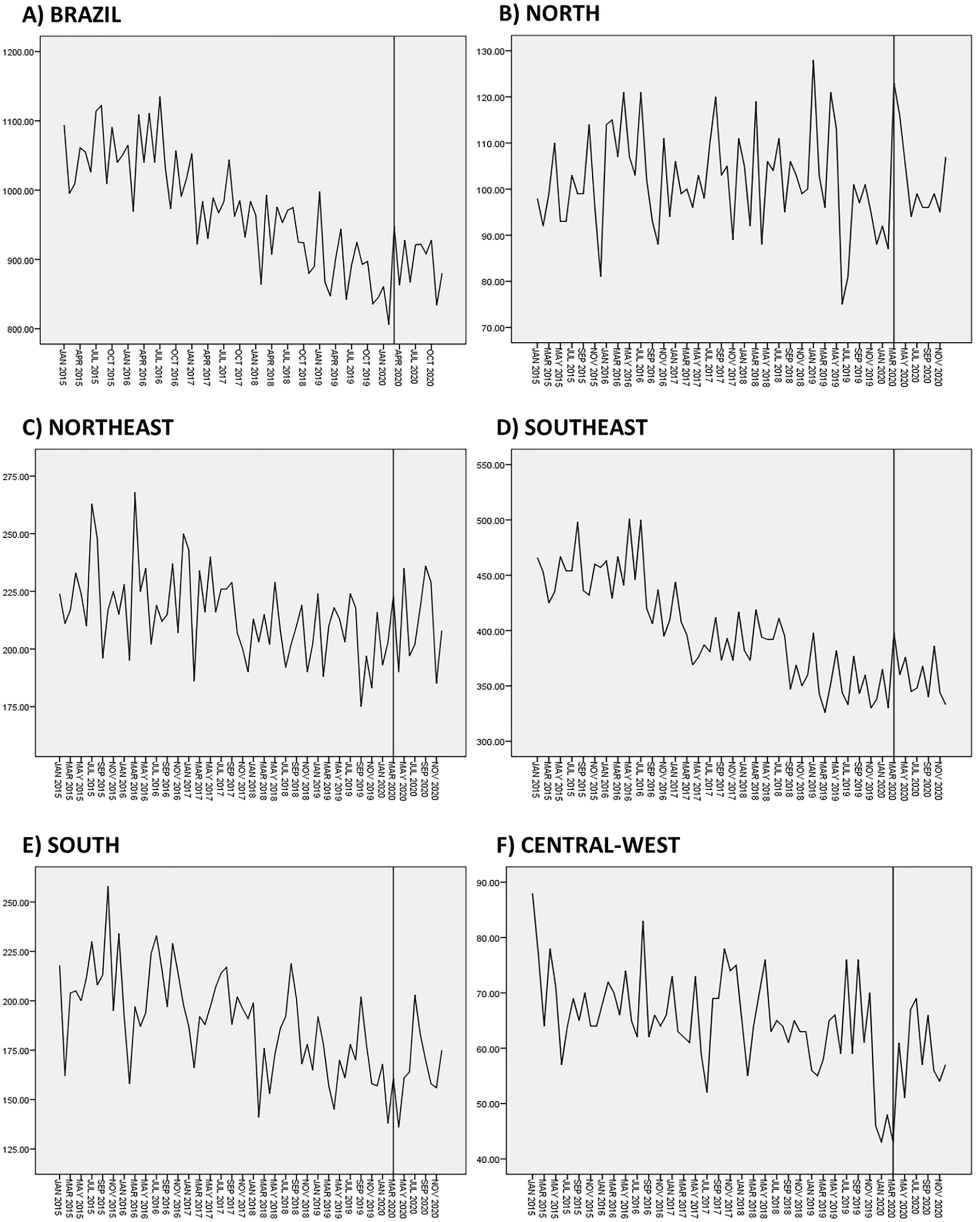
Interrupted time series analysis of mortality from AIDS in Brazil in 2020: (**A**) number of AIDS deaths in Brazil; (**B**) number of AIDS deaths in the North region of Brazil; (**C**) number of AIDS deaths in the Northeast region of Brazil; (**D**) number of AIDS deaths in the Southeast region of Brazil; (**E**) number of AIDS deaths in the South region of Brazil; and (**F**) number of AIDS deaths in the Central-West region of Brazil. The line that cuts each time series indicates the intervention point, that is the onset of the COVID-19 pandemic in Brazil in March 2020.

To corroborate the findings from the time trend analyses, an interrupted time series analysis was applied to determine whether the onset of the COVID-19 pandemic impacted the total number of AIDS deaths in Brazil in 2020. A non-stationary trend and a progressive reduction in AIDS mortality were observed in the last five years before the pandemic in Brazil. However, an increasing trend was noted after the onset of the pandemic in March 2020 (stationary R2 = 0.653; normalized BIC = 7.56; significance = 0.882; ARIMA estimate = 57.49; p-value = 0.002; Fig. 3A). This pattern was also observed in the Southeast (Fig. 3D) and South regions (Fig. 3E). Conversely, the North, Northeast, and Central-West regions exhibited a stationary trend before the COVID-19 pandemic, shifting to an increasing trend afterward (Fig. 3B–F).

**Figure 4:**
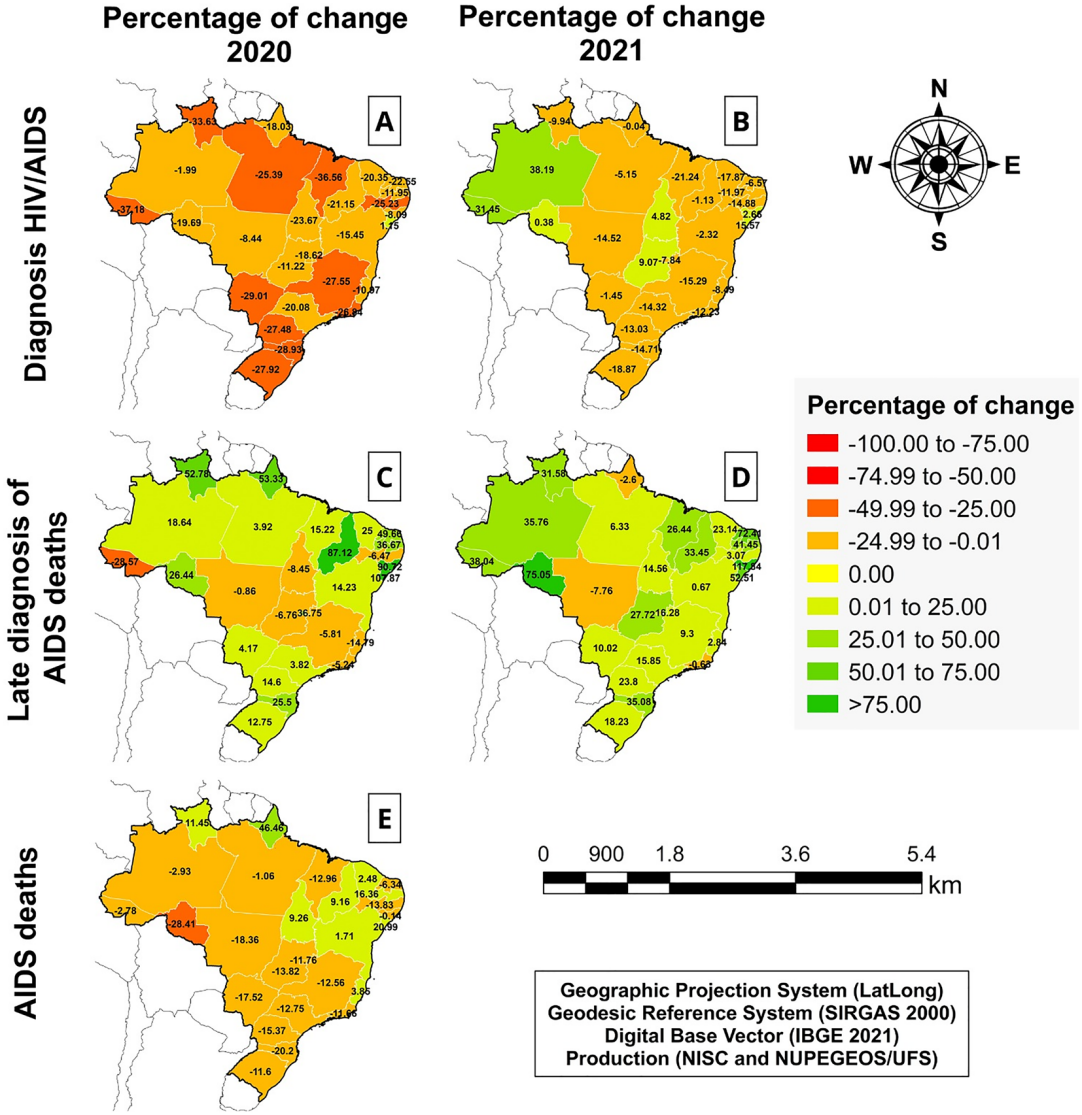
Spatial distribution of percentage change in key indicators of HIV/AIDS diagnosis and related mortality in Brazilian states during 2020 and 2021. (**A**) Number of HIV/AIDS cases in Brazil in 2020; (**B**) Number of HIV/AIDS cases in Brazil in 2021; (**C**) Late diagnosis AIDS deaths in Brazil in 2020; (**D**) Late diagnosis AIDS deaths in Brazil in 2021; (**E**) Total AIDS death in Brazil in 2020. QGis *3.20.4* (https://qgis.org/pt_BR/site/).

Concerning the spatial distribution of HIV/AIDS cases in Brazil (Fig. 4A), most states (n = 11) presented a percentage reduction of − 25 to − 49.99% during 2020. Meanwhile, although some states (n = 9) showed an increase in the percentage of HIV/AIDS cases in 2021, the majority still exhibited a percentage reduction, ranging from − 0.1 to − 24.99% (Fig. 4B).

In contrast, nine states experienced a percentage increase (ranging from 0.01 to 25%) in late diagnosis AIDS deaths during 2020 in Brazil (Fig. 4C). Additionally, ten states displayed a percentage change above 25% during the first year of the pandemic. The 2021 data revealed that 11 states had a percentage increase ranging from 0.01 to 25% in late diagnosis AIDS deaths (Fig. 4D). Notably, 12 states continued to show a percentage increase above 25% in 2021. Lastly, 19 states reported a percentage reduction ranging from -0.1% to -49.99% in the total number of AIDS deaths in Brazil during 2020 (Fig. 4E).

## Discussion

To the best of our knowledge, this is the first study to assess the impact of the COVID-19 pandemic on HIV/AIDS diagnosis and related mortality in all Brazilian states during 2020 and 2021. The analyses revealed that the total number of HIV/AIDS cases in Brazil decreased by 22.4% in 2020 and by 9.8% in 2021. Similarly, there was a decrease in overall AIDS deaths in Brazil in 2020 (− 9.4%) and a significant increase in the percentage of late diagnosis AIDS deaths in both 2020 (6.9%) and 2021 (13.9%). Remarkably, some states reported percentage increases exceeding 87% during both years. Overall, these results underscore the severe impact of the COVID-19 pandemic-related actions on HIV/AIDS control strategies, indicating a concerning and insidious scenario for the disease’s evolution in Brazil.

The implementation of emergency measures to control the spread of SARS-CoV-2 and reduce the risk of public health collapse negatively impacted various disease control programs, particularly those affecting tropical regions and vulnerable populations^2,14,16,19,36^. In this context, an assessment of the pandemic’s impact revealed that HIV/AIDS programs in many countries were affected in various ways: HIV staff were reassigned to support COVID-19 control, laboratory personnel were redirected for SARS-CoV-2 testing, HIV clinics were repurposed for COVID-19 services, there was limited availability of antiretrovirals (ARVs), interruptions in the HIV-related commodities supply chain, and fear of COVID-19 infection deterred people from visiting clinics^37^.

According to Bigoni and colleagues^38^, lockdown and other COVID-19 countermeasures resulted in decreases in diagnostic procedures (− 28.9%), physician appointments (− 42.5%), screenings (− 42.6%), and other medical procedures (− 15.5%), with a gradual reduction observed in every quarter of 2020. The country experienced a quarter-over-quarter reduction in healthcare services, with the most significant declines in exams, medical consultations, highly complex surgeries, transplants, and low- to medium-complexity surgeries. Surgeries and screening procedures were among the most affected, with most states experiencing a reduction of more than 50% throughout 2020.

Concurrently, the study by Villela and colleagues^39^ noted that mortality associated with COVID-19 was highest in Brazil’s Northern region (mainly in the state of Amazonas) and in two Northeastern states (Ceará and Pernambuco). They suggested that the variance between regions might have been influenced by the different degrees of restrictive measures adopted by the governors of Brazilian states. Consequently, scientists have been reporting that these measures will severely affect the goals of controlling HIV/AIDS worldwide, particularly in middle- and low-income countries^5,9,40^.

UNAIDS and WHO estimates indicate a significant reduction in HIV testing and fewer people starting antiretroviral therapy (ART), with a projected increase in new infections and AIDS-related deaths. It is estimated that between 120,000 and 290,000 new infections and 70,000 to 150,000 deaths will occur over two years, coinciding with the period of COVID-19 disruption. Moreover, discontinuation of HIV treatment during the coronavirus pandemic could result in over 500,000 unnecessary deaths^4^. Delays in AIDS diagnosis can also impact overall health, increasing the risk of developing AIDS-related complications and the likelihood of transmitting the virus to others^41^.

In 2014, UNAIDS launched the 90:90:90 initiative (90% of HIV infections diagnosed, 90% of AIDS cases on ART, and 90% under ART virally suppressed), aiming for achievement by 2020 (the goal is 95:95:95 by 2030)^12,13^. However, the global percentages in 2019 were 81%, 82%, and 88%, respectively, and it is crucial to note that global summaries mask regional differences. The epicenter of the pandemic remains in East and Southern Africa, accounting for 54% of all HIV-infected people, 43% of new HIV infections, and nearly 50% of related deaths^20^.

A study by Matsuda and colleagues^37^ found a significant reduction in the number of HIV tests performed during the pandemic in a Southeastern Brazilian city. Furthermore, the pandemic has significantly impeded progress towards the 90–90-90 HIV targets globally^42^. The delay in HIV diagnosis could lead to an increase in patients diagnosed with AIDS-related immunosuppression, as well as an increase in deaths.

Regional differences in HIV control were already evident in Brazil before the COVID-19 pandemic^37^. The South and Southeast regions, historically the epicenters of Brazil’s HIV pandemic, showed decreasing trends in HIV/AIDS diagnoses and AIDS mortality rates from 2015 to 2019. Other regions maintained stable trends during this period. However, stable trends were observed across Brazil and its regions during 2020 and 2021.

According to the Brazilian Ministry of Health, in 2020, the Northeast region had the highest number of AIDS diagnoses in Brazil (about 39%). In early 2021, the situation remained concerning, with an increase in AIDS cases in parts of the Northeast, particularly in the state of Bahia (4919 registered cases)^43^. The COVID-19 pandemic has exacerbated social and economic inequalities, impacting key HIV populations such as men who have sex with men, transgender people, sex workers, and drug users^41^. These groups, already facing barriers to healthcare access, likely encountered greater difficulties in maintaining HIV treatment during the pandemic^12^.

This pattern is mirrored in the USA, the high-income nation most heavily affected by HIV^12^. In 2018, a total of 37,881 new HIV infections were reported, with regional disparities; the South had twice the rate of new infections as the Midwest. Additionally, disparities persist along ethnic lines, with AIDS rates among blacks and African Americans eight times higher than among whites^20^. Similarly, data from South Africa reflect the impact of COVID-19 on HIV, evidenced by reduced testing, including for tuberculosis^4,6,8,44^. These findings highlight the importance of considering socioeconomic disparities in regional HIV/AIDS control efforts.

The analyses presented here revealed a significant increase in the number of late diagnosis AIDS deaths in 2020 and 2021, with some states reporting increases over 87%. This data is particularly alarming as it pertains to patients who had not been tested for or were unaware of their HIV serologic status, and who were only diagnosed with AIDS at the time of death. These staggering numbers observed in 2020 and 2021 are likely linked to COVID-19 clinical complications in AIDS patients with immunodeficiency not receiving ART treatment.

There are various profiles of people living with HIV, and the impact of COVID-19 may differ among them^5^. Despite some contradictory data, it is evident that SARS-CoV-2 has had a detrimental impact on people living with HIV. Previous studies have shown that comorbidities increase the risk of severe clinical forms of COVID-19, including immunosuppression, cardiovascular disease, cancer, and tuberculosis^20,44,45^.

Before the advent of COVID-19, HIV/AIDS and tuberculosis were considered the two most lethal infectious diseases globally^46^. UNAIDS estimated that in 2021, one in three AIDS-related deaths was due to tuberculosis^43^. Moreover, the occurrence of COVID-19 in patients co-infected with HIV/AIDS and tuberculosis can result in more severe clinical outcomes^44^. All three diseases can significantly affect the lungs, often leading to a cytokine storm, immunosuppression, and respiratory failure. Co-infections, coupled with SARS-CoV-2, contribute substantially to the morbidity and mortality of COVID-19, with these patients being four times more likely to develop post-COVID-19 conditions.

This situation poses a major medical and socioeconomic concern, particularly in regions with a high burden of tuberculosis and HIV/AIDS, such as in Africa, Asia, and South America^46,47^. Disparities in HIV/AIDS control programs were evident even before the COVID-19 pandemic, reflecting longstanding inequality. The international response to AIDS was initially slow, with a global program only launched six years after the onset of the pandemic. Many African countries still lack comprehensive plans to address a global AIDS pandemic^4^.

Brazil had been experiencing a gradual reduction in the number of HIV/AIDS infections and deaths in recent years, with a downward or stable trend^43^. However, in 2020, almost all states (except Sergipe) observed a sudden reduction in HIV/AIDS diagnoses. Conversely, some states, such as Alagoas, recorded a significant increase in AIDS-related deaths, exceeding 100%. This underscores the severity of the situation and the urgent need to reinforce HIV/AIDS strategic plans. The sudden decrease in HIV/AIDS diagnoses is likely due to difficulties in accessing health services, fear of testing, and limited care during COVID-19 social distancing measures. Mobility restrictions in several Brazilian states may have impeded HIV diagnosis, as people were discouraged or unable to travel to locations offering HIV testing and treatment^36,48^.

Therefore, the apparent reduction in mortality should not be misinterpreted as an improvement in HIV epidemic control in the country. While there has been a reduction in HIV/AIDS deaths in most Brazilian states, deaths related to late AIDS diagnosis have significantly increased. It is probable that fewer people were being tested, and the actual number of HIV cases is likely higher, mainly due to the disruption of national health surveillance activities.

Additionally, the deterioration of AIDS indicators may reflect the actions of the Brazilian administration during the 2018–2022 period towards disease control programs. In these years, Brazil experienced a disruption of strategies and advances previously achieved in the Unified Health National System, including the implementation of new treatments, investments in antiretroviral therapy, expansion of health services (testing and counseling centers), and prevention campaigns^49^. The AIDS Department of the Brazilian Ministry of Health underwent significant organizational restructuring. This Program, a world reference until mid-2018 for offering free universal treatment to all Brazilians, faced severe resource reductions, researchers lost funding, and health professionals were reallocated or dismissed from health services^49^. The repercussions of this strategy are evident in both the COVID-19 pandemic scenario and the mortality rates due to late AIDS diagnosis in Brazil. These developments raise a critical question: will Brazil achieve the WHO’s 2030 goals for disease control? One of the major challenges for Brazil's public health system in the post-COVID scenario is the revitalization and restoration of health actions and services that were largely suspended during the two years of the pandemic.

While the epidemiological situation of COVID-19 is increasingly under control, it is clear that the HIV/AIDS pandemic should not be overlooked. These factors make the 2030 AIDS targets a significant challenge^42^. Unfortunately, the 2022 database is not yet available to continue the analysis of HIV/AIDS mortality and deaths due to late AIDS diagnosis. However, the trends observed during the first 2 years of the COVID-19 pandemic present a concerning scenario for HIV/AIDS in Brazil and highlight the urgent need to develop and reinforce control strategies to improve the situation.

Lastly, this study has limitations that must be acknowledged. The ecological assessment of secondary data from SINAN, SIM, and SISCEL opens the possibility for biases, such as underreporting or overreporting of cases in certain regions or states. Additionally, there might be delays in data registration within the system. Despite these limitations, the findings provide important insights into the impact of the pandemic on decision-making processes related to HIV/AIDS in Brazil.

## Conclusions

The analyses revealed a reduction in the total number of HIV/AIDS cases in Brazil during the first 2 years of the COVID-19 pandemic. However, during the same period, there was a significant increase in the percentage of late diagnosis AIDS deaths, with some states experiencing increases of more than 87%. These findings underscore the profound impact on HIV/AIDS control strategies and reveal a worrisome scenario for achieving HIV/AIDS control goals in Brazil. Given these circumstances, it is imperative for health authorities to urgently reorganize and strengthen infectious disease control programs. Otherwise, the world may encounter unprecedented challenges in achieving various public health goals. The current crisis presents an opportunity to institutionalize and fund more community-led responses to HIV/AIDS. Implementing funding strategies to mitigate the impact of COVID-19 on HIV/AIDS programs, especially in middle- and low-income countries, is essential. The HIV/AIDS pandemic is far from resolved, coupled with the ongoing need to address the persistence of COVID-19 as an endemic threat. It is possible that common lessons learned from both pandemics could be leveraged to control both diseases. Finally, effective global health approaches must take into account the impact of pandemics on other infectious diseases and the necessity of sustaining comprehensive public health measures.

## Data Availability

The data sets related to leprosy analyzed and that support the results of this study are available on the website of the Department of Informatics of the Unified Health System (DATASUS) and registered in the Notifiable Diseases Information System (SINAN): http://www.portalsinan.saude.gov.br/; the Mortality Information System (SIM): https://opendatasus.saude.gov.br/dataset/sim; and the Control System for Laboratory Tests (SISCEL): https://www.gov.br/aids/pt-br/indicadores-epidemiologicos/sistemas-de-informacao/siscel. For the construction of spatial analysis maps, the cartographic base of Brazil was used, available in the electronic database of the Instituto Brasileiro de Geografia e Estatística (IBGE): https://www.ibge.gov.br/geociencias/organizacao-do-territorio/malhas-territorio/15774-malhas.html?=&t=o-que-e.
